# The Role of Descending Pain Modulation in Chronic Primary Pain: Potential Application of Drugs Targeting Serotonergic System

**DOI:** 10.1155/2019/1389296

**Published:** 2019-12-17

**Authors:** Zhuo-Ying Tao, Pei-Xing Wang, Si-Qi Wei, Richard J. Traub, Jin-Feng Li, Dong-Yuan Cao

**Affiliations:** ^1^Key Laboratory of Shaanxi Province for Craniofacial Precision Medicine Research, Research Center of Stomatology, Xi'an Jiaotong University College of Stomatology, 98 West 5th Road, Xi'an, Shaanxi 710004, China; ^2^Department of Oral and Maxillofacial Surgery, Xi'an Jiaotong University College of Stomatology, 98 West 5th Road, Xi'an, Shaanxi 710004, China; ^3^Department of Neural and Pain Sciences, School of Dentistry; Center to Advance Chronic Pain Research, University of Maryland Baltimore, 650 W Baltimore St., Baltimore, MD 21201, USA

## Abstract

Chronic primary pain (CPP) is a group of diseases with long-term pain and functional disorders but without structural or specific tissue pathologies. CPP is becoming a serious health problem in clinical practice due to the unknown cause of intractable pain and high cost of health care yet has not been satisfactorily addressed. During the past decades, a significant role for the descending pain modulation and alterations due to specific diseases of CPP has been emphasized. It has been widely established that central sensitization and alterations in neuroplasticity induced by the enhancement of descending pain facilitation and/or the impairment of descending pain inhibition can explain many chronic pain states including CPP. The descending serotonergic neurons in the raphe nuclei target receptors along the descending pain circuits and exert either pro- or antinociceptive effects in different pain conditions. In this review, we summarize the possible underlying descending pain regulation mechanisms in CPP and the role of serotonin, thus providing evidence for potential application of analgesic medications based on the serotonergic system in CPP patients.

## 1. Introduction

Chronic pain is a severe problem for the general population worldwide. Though pain itself does not cause death immediately, long-term suffering from pain exerts a negative impact on both work and living quality of patients. Some pain conditions are evoked by specific tissue damage which can be cured by cause-based treatment. However, others are absent of pathological alterations, such as fibromyalgia syndrome (FMS), irritable bowel syndrome (IBS), or temporomandibular disorder (TMD) [1, 2]. During the past few decades, many different concepts have been used to describe these diseases, including functional somatic syndromes, somatoform pain disorders, and functional pain syndromes [3–5]. All these concepts regard “chronic pain” as a symptom only. Recently, the International Classification of Diseases-11 (ICD-11) has raised a new concept “chronic primary pain (CPP)” for these diseases, defining it as pain in one or more anatomical regions that (1) persists or recurs for over 3 months; (2) is associated with significant emotional distress or functional disability (interference with activities of daily life and participation in social roles); and (3) cannot be better accounted for by another chronic pain condition [6]. CPP includes a constellation of diseases with medically unexplained pain and disability which cause functional impairment or disruption of daily activities, namely, chronic widespread pain (e.g., FMS), complex regional pain syndromes, chronic primary headache and orofacial pain (e.g., chronic migraine or TMD), chronic primary visceral pain (e.g., IBS), and chronic primary musculoskeletal pain (e.g., nonspecific low back pain). This concept distinguishes functional pain without diagnostic laboratory tests or convincing medical explanations from pain secondary to underlying diseases. The most prominent progress of the new definition is that ICD-11 has incorporated “chronic pain” as a disease itself [7], which may contribute to the deeper understanding of pain pathophysiology and management.

CPP is a more severe problem in medical practice nowadays, not only because of its high prevalence (the general population prevalence for IBS is 11.2% [8], for FMS is 2.1% [9], and for TMD is 5-10% [10]) but also due to the high related consumption of health care resources [11, 12]. However, due to the lack of a definite pathology and the uncertainty of etiology and underlying mechanisms, the management of CPP is still poor. Patients often suffer from two or more diseases of CPP, resulting in comorbid or chronic overlapping pain conditions (COPC) [13, 14]. Therefore, most patients seek treatment from physicians in different departments of hospitals, e.g., gastroenterologists, rheumatologists, dentists, neurologists, cardiologists, gynecologists, and otorhinolaryngologists. Eventually, patients do not receive fulfilling therapy, resulting in not only considerable cost in money and time but also intractable pain and anxiety. Hence, the development of new therapeutics for the pain management of CPP has become a critical need.

The descending pain system plays an important role in different pain conditions, and it is well recognized that descending control can be either facilitatory or inhibitory even though it cannot be fully dissociated anatomically [15]. The descending pain facilitatory system consists of the anterior cingulate cortex (ACC), the rostral ventromedial medulla (RVM), and the dorsal reticular nucleus of the medulla [16–18], while the descending inhibitory system includes the periaqueductal gray (PAG), RVM, and caudal lateral ventrolateral medulla (VLM) [19, 20]. The descending control modulates pain circuitry at multiple levels, and numerous findings have identified that the imbalance of descending pain modulation favoring pain facilitation contributes to the promotion and maintenance of chronic pain [21].

The serotonergic system plays a critical role in the modulation of nociception mainly through descending pain circuits, during which neural plasticity changes between different regions of the brain [22]. Serotonin (5-hydroxytryptamine (5-HT)), an important neurotransmitter, exhibits its effect via activating different receptor subtypes [23, 24]. Besides 5-HT, some antidepressants, including tricyclic antidepressants (TCAs), selective serotonin reuptake inhibitors (SSRIs), and serotonin-norepinephrine reuptake inhibitors (SNRIs), can also exert an impact on pain perception through the 5-HT system [25]. In conditional knockout mice lacking central serotonergic neurons, SSRI-mediated analgesia was greatly reduced, suggesting the involvement of serotonergic neurons in the pain pathways [26]. Recently, studies on the effects of the descending pain system in CPP, such as IBS and FMS, have been performed, identifying the potential role of its therapeutic value [27, 28]. Here, we review (1) the descending pain mechanisms mediated by the 5-HT system, focusing on CPP, including FMS, IBS, TMD, and chronic primary headache; (2) the clinical application of current and potential medicines targeting the serotonergic system in pain management; and (3) their prospects in CPP.

## 2. Chronic Primary Pain

### 2.1. Characteristics

Clinical studies indicate the diseases comprising CPP have many characteristics in common. First, chronic, diffuse, and intractable pain at different locations from head to limbs can be the most obvious symptom and it is always the main complaint of the patients. Second, functional disturbance in different organ systems (e.g., palpitation, dizziness, constipation or diarrhea, movement, insomnia, fatigue, or exhaustion) can be another frequent bodily complaint [29]. Third, CPP is more prevalent in women. Female patients are twice as likely to seek treatment for TMD and IBS compared to male patients [30, 31], and women outnumber men by an average of 3 : 1 in FMS patients from epidemiological studies [32]. Fourth, CPP patients are likely to have a history of physical or sexual abuse or childhood adversity, and the symptoms can be exacerbated by stress [33, 34]. Fifth, these patients tend to suffer multiple conditions of CPP and overlap with psychiatric disorders such as anxiety and depression [35, 36].

CPP has a high prevalence worldwide, and the therapeutic treatment of CPP is unsatisfactory due to the unknown etiology. Both physicians and patients may feel confused about medically unexplained pain and are unfamiliar with how to give/obtain effective treatment since conventional medical therapy sometimes seems to be of no significant effect on these diseases. Some physicians even think the symptoms of CPP patients are less severe than they are reported when the symptoms cannot be well explained by contemporary medicine or definite pathology, leading to trust crises between physicians and patients [37]. Thus, CPP patients have more outpatient visits with associated expenditure on health care and are more likely to request/receive painkillers [11, 12].

### 2.2. Possible Underlying Mechanism: Dysfunction of Descending Pain Facilitatory and Inhibitory System

Two or more diseases of CPP tend to occur in one patient clinically. This may attribute to the consistent central mechanisms of CPP [7]. Therefore, the pain management of CPP may be similar. Although macroscopic peripheral damage is generally absent in CPP, there are some common pathophysiologic findings in the nervous system including abnormalities of the hypothalamus-pituitary-adrenal (HPA) axis, the autonomic nervous system, and sensory processing (central sensitization and a lack of descending inhibitory activity) [38–40]. In recent years, studies have identified an essential role of the descending pain modulation and its alterations in neuroplasticity in CPP. A systematic review on structural and functional brain magnetic resonance imaging (MRI) of FMS patients showed that the gray volume of the ACC decreased, but its functional activities increased and functional connectivity between brain regions in the descending pain inhibitory system decreased compared to healthy controls [41]. A clinical study found a correlation between decreased gut permeability and increased functional and structural connectivity of the right amygdala in moderate-to-severe IBS patients, while decreased gut permeability was associated with increased connectivity between the default mode network (DMN) and PAG in healthy controls [42]. Women with primary dysmenorrhea, a classified chronic pelvic pain syndrome, showed increased gray matter volume in the hypothalamus, hippocampus, PAG, and ACC [43], indicating reactive pain modulation. The high clinical overlap between painful TMD and headache disorders may attribute to the impairment of the descending inhibitory pain pathways [44]. These results suggest that the hyperactivity of descending pain facilitatory and/or the impairment of descending inhibitory system may contribute to CPP, providing a possible explanation of the overlap of pain in CPP patients and also a prospective therapeutic avenue for pain alleviation targeting the descending pain modulation.

## 3. The Role of Serotonergic System in Descending Pain Modulation

Maintaining a stable baseline of pain perception and processing can be attributed to the normal function of descending pain facilitatory and inhibitory systems. Abnormalities of these systems can lead to analgesia or hyperalgesia [45]. Many different neurotransmitters are involved in the occurrence and development of pain when disturbing this balanced state [46]. Among them, it has been well established that the descending serotonergic pathways can exert either a pronociceptive or antinociceptive impact, depending on different pain states and the multiplicity of subtype receptors activated [23, 47, 48]. 5-HT is widely distributed in different systems, including the nervous, gastrointestinal, and cardiovascular systems, and modulates a considerable collection of physiological and pathological conditions, including pain, sleep regulation, aggression, feeding, anxiety, and depression [49].

The abnormality of 5-HT signaling in different pain states has been identified in both basic research and clinical investigation and may be a possible and potential explanation for some diffuse pain states. In some neuropathic pain models, the baseline level of 5-HT in the spinal cord was decreased [50, 51], while in formalin or carrageenan induced-inflammatory pain models, the release of 5-HT in the dorsal raphe increased [52]. In FMS patients, the plasma 5-HT level decreased and its metabolite, 5-hydroxyindolacetic acid (5-HIAA) in the cerebrospinal fluid, also decreased [53]. These results demonstrate both a facilitatory role and an inhibitory role of 5-HT in the central nervous system (CNS), but the differences between these two opposing effects were not clear in these studies.

More recent studies focused on 5-HT receptor subtypes, which differ in structure, action, and localization in both the central and peripheral nervous systems. Most of these receptors are widely expressed in the spinal dorsal horn, where nociceptive information is relayed from primary afferent fibers and modulated by descending fibers prior to being transmitted to supraspinal sites [49]. Seven families of 5-HT receptors (5-HT_1-7_) comprising 14 distinct receptor subtypes have been identified, including 5-HT_1A-B_, 5-HT_1D-F_, 5-HT_2A-C_, 5-HT_3_, 5-HT_4_, 5-HT_5A-B_, 5-HT_6_, and 5-HT_7_ [54]. These receptors can be divided into two separate protein superfamilies: ligand-gated ion channel receptors (5-HT_3_) and the G protein-coupled 7-transmembrane receptor superfamily (the rest of other 13 subtypes) [54]. With the synthesis and use of relatively selective agonists and antagonists for these subtypes, different animal models have been used to investigate the downstream effects of 5-HT/receptor signaling leading to effective or potential drugs in clinical application for numerous diseases including psychiatric disorders, pain conditions, gastrointestinal diseases, obesity, nausea, and vomiting. As for the function of 5-HT receptors in descending pain modulation, the activation of the 5-HT_1A_, 5-HT_1B_, 5-HT_1D_, 5-HT_5A_, and 5-HT_7_ receptors is prone to exert an antinociceptive effect, whereas the 5-HT_2B_ and 5-HT_3_ receptors tend to contribute to the promotion of nociception [23, 47, 48, 55–58]. However, there are still controversy and uncertainty over the role of these receptors in mediating nociceptive processing due to the location of the receptors, routes of drug administration, concentration and duration effects of agonists or antagonists, and even pain types in different studies [59]. In addition, some antidepressants like TCAs, SNRIs, and SSRIs can also influence the descending pain modulation system by increasing 5-HT at the synaptic junction. The detailed function and present/future clinical application of 5-HT receptor agonists/antagonists and reuptake inhibitors affecting the serotonergic system in pain modulation will be reviewed and discussed below.

### 3.1. 5-HT_1_ Receptors

The 5-HT_1_ receptor family includes 5-HT_1A_, 5-HT_1B_, 5-HT_1C_, 5-HT_1D_, 5-HT_1E_, and 5-HT_1F_ receptors [54]. After the 5-HT_1C_ receptor was cloned and its characteristics identified, it was reclassified as a subtype of the 5-HT_2_ receptor family, the 5-HT_2C_ receptor [60].

The role of the 5-HT_1_ receptor family except 5-HT_1E_ in pain has been long studied both together and separately. Among them, the 5-HT_1A_ receptor has been extensively investigated for its analgesic effect in multiple pain states, including neuropathic, inflammatory, and visceral pain [61–63]. The activation of the 5-HT_1A_ receptor inhibits adenylyl cyclase thus reducing the intracellular concentration of cyclic adenosine monophosphate (cAMP), and subsequently, K^+^ channels open and Ca^2+^ channels close, resulting in an inhibition of neuronal firing [64]. The analgesic effect of 5-HT_1A_ receptors has been widely recognized after 5-HT_1A_ receptor-deficient (knockout) mice were established [65]. These mice have a higher nociceptive response in a hot plate test [65]. In the spinal cord, the activation of 5-HT_1A_ receptors can inhibit glutamate release, reinstate GABA-dependent inhibition, and inhibit phosphorylation of Ca^2+^/calmodulin-dependent protein kinase II (CaMKII) to reduce pain transmission [66–68]. Supraspinally, 5-HT_1A_ receptors exert an antinociceptive impact via descending pain inhibitory pathway. 5-HT_1A_ receptors may contribute to inhibitory modulation of glutamate release in the ACC [69] and the inhibitory action of the GABAergic interneurons in the ventrolateral orbital cortex [70]. Glutamate administration in the central amygdala (CeA) produced bidirectional effects: increased hypersensitivity that was reversed by a spinal 5-HT_3_ receptor antagonist and decreased hypersensitivity that was blocked by a spinal 5-HT_1A_ receptor antagonist, indicating a CeA-spinal descending pathway in pain modulation [71]. Somatosensory cortex (S2) stimulation caused antinociception in a spinal nerve ligation (SNL) model, and this effect was prevented by chemically activating 5-HT_1A_ receptors in the RVM or blocking 5-HT_1A_ receptors spinally, illustrating that S2 stimulation-induced analgesia in neuropathic pain is mediated by medullospinal descending serotonergic pathways [63]. In visceral pain, a clinical trial showed that tandospirone citrate (a partial agonist of 5-HT_1A_ receptors) had benefits in suppressing the abdominal symptoms of patients with functional dyspepsia [62], which was consistent with animal experiments [66]. Interestingly, in contrast to morphine, the analgesic effect associated with 5-HT_1A_ receptor agonists increases with repeated or chronic treatment [72, 73], suggesting a potential for their application in intractable pain management.

Because of the highly homologous structures, 5-HT_1B/1D/1F_ receptors have similar functions in nociceptive information processing, especially in migraine. Triptans, the agonists of 5-HT_1B/1D_ receptors, are the first-line acute therapy for patients who suffer from moderate-to-severe migraine attacks through acting on cerebral blood vessels to constrict vessels selectively and reduce neurogenic inflammatory mediators including calcitonin gene-related peptide [74]. Lasmiditan, a new selective 5-HT_1F_ receptor agonist and a promising drug in migraine, controls the symptoms via inhibiting dural plasma protein extravasation and decreasing c-Fos in the trigeminal nucleus caudalis without vasoconstrictive activities in peripheral tissues [75]. Some studies have identified the neural mechanisms of triptans in migraine besides vascular effects. Specifically, sumatriptan inhibited GABAergic and glutamatergic synaptic transmission through 5-HT_1B/D_ receptors in the PAG [76], induced dose- and time-related blockade of visceral pain with intra-RVM administration [77], and reduced the release of glutamate presynaptically in the substantia gelatinosa of the spinal trigeminal nucleus pars caudalis [78], indicating that descending pain inhibitory system may be an additional potential site of action for the modulation of pain by 5-HT_1B/1D_ receptors. Numerous behavioral or electrophysiological studies have already confirmed an analgesic role of the 5-HT_1B/1D_ receptor at the spinal level [79, 80]. A recent study focused on the downstream molecular mechanism of 5-HT_1B_ receptors in the spinal cord. Specifically, 5-HT_1B_ receptors activated phospholipase D1 which further activated phosphatidylinositol 4-phosphate (PIP) 5-kinase and PIP 2, leading to an allosteric enhancement of transient receptor potential melastatin 8 (TRPM8) [81]. However, there is no study on the PAG-RVM-spinal pathway or upstream mechanisms of these receptors. Further studies are needed to enrich the mechanism of 5-HT_1B/1D_ receptors in descending inhibitory pain pathways, in order to support the application of 5-HT_1B/1D_ receptor agonists in pain conditions.

### 3.2. 5-HT_2_ Receptors

The 5-HT_2_ receptor family contains three subtypes, 5-HT_2A_, 5-HT_2B_, and 5-HT_2C_ receptors [49]. 5-HT_2A_ receptors are expressed in descending pain modulation pathways, including the nucleus raphe magnus (NRM), ventrolateral PAG (vlPAG), and the spinal dorsal horn, and also in reticular formation, thalamus, and limbic structures [82, 83]. The distribution of 5-HT_2B_ receptors is restricted to a few brain regions, including the cerebellum, lateral septum, dorsal hypothalamus, medial amygdala, and spinal cord [54]. 5-HT_2C_ receptors are widely distributed in the locus coeruleus (LC), PAG, retrorubral area, substantia nigra pars compacta, ventral tegmental area, parabigeminal nucleus, basal nucleus, and laterodorsal tegmental nucleus [59].

Regarding the 5-HT_2_ receptor family, most of the studies on pain modulation are focused on 5-HT_2A_ and 5-HT_2C_ receptors, with minimal studies examining 5-HT_2B_ receptors. Furthermore, whether central 5-HT_2A/2C_ receptors are pronociceptive or antinociceptive is controversial. In a recent study, blocking 5-HT_2A/2C_ receptors in both the NRM and the gigantocellularis/paragigantocellularis pars reticular nuclei (Gi/PGi) reduced unconditioned fear-induced antinociception, suggesting that 5-HT_2A/2C_ receptors in the Gi/PGi complex and NRM are critically recruited and participate in hypoalgesia during the panic-like emotional behavior [84]. Also, 5-HT_2A/2C_ receptors in the dorsomedial PAG (dmPAG), vlPAG, dorsal raphe nucleus (DRN), and LC play a critical role in the elaboration of postictal antinociception [85–87]. However, a recent study found that increased expression of 5-HT_2C_ receptors in non-GABAergic cells of the basolateral nucleus of amygdala (BLA) in rats with neuropathic pain and knockdown of 5-HT_2C_ receptor in the BLA inhibited pain-related behaviors [88]. Another study reported that 5-HT_2C_ in the amygdala reduced the effectiveness of SSRIs in inhibiting pain-related emotional-affective behaviors [89], indicating the participation of amygdala 5-HT_2C_ receptors in pronociception. At the supraspinal level, 5-HT_2A/2C_ receptors are more likely to contribute to antinociception, whereas they participate in either analgesia or hyperalgesia in the spinal cord from different reports. Activation of spinal 5-HT_2A/2C_ receptors increased the pain-related behavioral responses in the early and late phases during formalin tests and also long-lasting hypersensitivity post formalin treatment [90, 91]. But in spinal nerve injury models, intrathecal administration of 5-HT_2A_ or 5-HT_2C_ receptor agonists produced antiallodynic effects [92, 93].

Further research has tried to investigate the contradictory results of 5-HT_2A_ receptors in pain modulation. A recent study found that the mechanical allodynia was related to sensitization, reduced dendritic arborization, and enhanced spine density exclusively in interneurons expressing the *γ* isoform of protein kinase C (PKC*γ*^+^) in a rat model of facial inflammatory pain. Blockade of 5HT_2A_ receptors abolished the facial mechanical pain and related changes in the morphology of PKC*γ*^+^ interneurons, suggesting that inflammation-induced pain contributes to morphological reorganization of PKC*γ*^+^ interneurons via 5-HT_2A_ receptor activation in pain circuitry [94]. Chemically activating 5-HT_2A_ receptors enhanced C-fiber-evoked dorsal horn potentials after SNL, which could be blocked by a metabotropic glutamate receptor 1 (mGluR1) antagonist, and 5-HT_2A_ receptors and mGluR1 were coexpressed in postsynaptic densities in dorsal horn neurons, confirming mGluR1 upregulation as a novel mechanism accounting for 5-HT_2A_ receptor-mediated pain facilitation [95]. However, an inhibitory role for 5-HT_2A_ receptors has also been identified. Activation of 5-HT_2A_ receptors hyperpolarized the reversal potential of inhibitory postsynaptic potentials (IPSPs), E_IPSP_ in spinal motoneurons, activated PKC, and increased the cell membrane expression of neuron-specific K^+^-Cl^–^ cotransporter (KCC2) after spinal cord injury in rats, indicating a PKC-dependent 5-HT_2A_-KCC2 pathway in neuropathic pain regulation [96]. Later, this pathway was confirmed to participate in other pain models, including thoracic hemisection, spared nerve injury, and incision pain [97, 98]. Another study showed that after activating 5-HT_2A_ receptors by intrathecal injection of a cell-penetrating peptidyl mimetic of the 5-HT_2A_ receptor C-terminus (TAT-2ASCV), the interaction between 5-HT_2A_ receptors and postsynaptic density protein-95 (PSD-95, one of the PSD-95/disc large suppressor/zonula occludens-1 domain containing proteins) was disrupted, followed by an anti-hyperalgesic effect in neuropathic pain of diabetic rats. Also, TAT-2ASCV enhanced SSRI-induced anti-hyperalgesia [99]. Recent studies identified this process in traumatic neuropathic pain and inflammatory pain models [100, 101], as well as the evidence that spinal GABA release and GABA_A_ receptor activation are involved in this process [101, 102]. Presently, a final conclusion of the facilitatory or inhibitory role of 5-HT_2A/2C_ receptors in pain-related circuits in the descending pain system is still not clear. Though some medications targeting 5-HT_2A/2C_ receptor subtypes have been applied in clinical practice, including blonanserin (5-HT_2A_ receptor antagonist) in schizophrenia [103] and lorcaserin (5-HT_2c_ receptor agonist) in obesity [104], more studies are needed to figure out the function and mechanism of 5-HT_2A/2C_ receptors in the descending pain modulation pathway.

As for 5-HT_2B_ receptors, limited studies have identified a pronociceptive role in pain control, supporting the prospect of 5-HT_2B_ receptor antagonists as painkillers in the future. In a SNL model, 5-HT_2B_ receptor expression increased in the ipsilateral dorsal spinal cord and dorsal root ganglia (DRG), and intrathecal administration of the 5-HT_2B_ receptor antagonist diminished both allodynia and 5-HT_2B_ expression after nerve injury [105]. Another study confirmed that 5-HT_2B_ receptor-mediated facilitation in neuropathic pain may contribute to the transient activation of the PKC*γ*/NMDA receptor pathway [106]. In visceral hypersensitive Wistar Kyoto rats, both peripheral and intracerebroventricular injection of RS-127445 (5-HT_2B_ receptor antagonist) reduced pain behaviors during noxious colorectal distension, providing a possible target for pain relief in gastrointestinal disorders such as IBS [107]. However, the present studies on 5-HT_2B_ receptor antagonists in pain alleviation are not yet adequate to organize preclinical or clinical studies investigating the efficacy and safety in human beings.

### 3.3. 5-HT_3_ Receptors

The 5-HT_3_ receptor is the only ligand-gated ion channel among all 5-HT receptor subtypes [54]. 5-HT_3_ receptor has two subunits (5-HT_3A_ and 5-HT_3B_) in rodents [108] and five subunits (5-HT_3A-E_) in human beings [109]. In the nervous system, functional 5-HT_3_ receptors can be formed from either five 5-HT_3A_ subunits (homopentameric) or a mixture of 5-HT_3A_ and one of the other four receptor subunits (heteropentameric) [110]. 5-HT_3_ receptors are highly expressed in the dorsal vagal complex of the brainstem, hippocampus, amygdala, superficial layers of the cerebral cortex, and spinal dorsal horn [54].

The role of 5-HT_3_ receptors in nociceptive processing in the descending pain pathway has been widely investigated through 5-HT_3_ receptor agonists (e.g., SR57227, YM-31636, and mCPBG) and antagonists (e.g., Y25130, granisetron, ondansetron, ramosetron, palonosetron, dolasetron, and tropisetron). Though there are some early studies which reported antinociceptive effects of 5-HT_3_ receptors [111, 112], it is now well recognized that 5-HT_3_ receptors play an important role in the descending pain facilitatory system based on the genetic studies clarifying that 5-HT_3_ receptors are involved in persistent, but not acute, pain. Specifically, in 5-HT_3_ knockout mice, a significant reduction of the second phase, but not the first phase, of pain behavior in the formalin test was reported compared to wild-type mice [65, 113]. Also, 5-HT_3_ receptors had no effect on acute pain produced by physiologically relevant stimuli and an antagonist did not change nociceptive thresholds when it was administered alone in wild-type mice without tissue injuries [113]. Recently, electrophysiological, molecular, biological, and behavioral studies have confirmed that the activation of 5-HT_3_ receptors in the spinal cord is involved in persistent pain, including chronic inflammatory pain [55], postoperative pain [17], visceral hypersensitivity [114], neuropathic pain [115], cancer pain [116], and opioid-induced hyperalgesia [117]. Specifically, systemic administration of the serotonin precursor producing visceral hypersensitivity and somatic analgesia could be attenuated by intrathecal treatment of ondansetron (5-HT_3_ receptor antagonist) [118], and spinal injection of ondansetron also exerted an analgesic impact in animal models of spinal nerve injury, cancer-induced bone pain, osteoarthritis, and hyperalgesia induced by morphine treatment [55, 115–117].

In addition to 5-HT_3_ receptors and spinal modulation of pain, the role of supraspinal regions modulating spinal 5-HT_3_ receptors have been examined. For example, intrathecal injection of ondansetron can reverse the mechanical hypersensitivity induced by low-dose administration of glutamate in the CeA [71]. Thermal hyperalgesia and tactile allodynia induced by microinjection of cholecystokinin (CCK) in RVM are attenuated by spinal administration of ondansetron [47], indicating descending control from the amygdala and RVM to facilitate pain via activating 5-HT_3_ receptors in the spinal cord. In a chronic postoperative pain model, increased 5-HT release and 5-HT_3_ receptor expression and activation of microglia were found in the L3 spinal dorsal horn. Intrathecal injection of Y-25130 could block pain behaviors and molecular changes. Meanwhile, in the RVM, P_2_X_7_ receptors on microglia were upregulated, microglia were activated, and 5-HT release increased. Microinjection of a P_2_X_7_ receptor antagonist into RVM reversed the pain behaviors and molecular changes as well. These results have suggested that P_2_X_7_ receptor activation in microglia in the RVM contributes to the enhancement of the RVM-spinal 5-HT system through actions of 5-HT_3_ receptors and hyperactivity of microglia at the spinal level [17]. Another study investigated the neuron-microglia-astrocyte-neuron circuit after activating 5-HT_3_ receptors in the spinal cord, explaining the interactions between 5-HT_3_ receptors and microglial activity in pain processing [119]. Specifically, intrathecal administration of the 5-HT_3_ receptor agonist SR57227 induced selective activation of 5-HT_3_ receptors in neurons which released the neuroactive substance fractalkine targeting its receptors on microglia. The activated microglia upregulated and released interleukin- (IL-) 18 to activate astrocytes which released IL-1*β* [119]. IL-1*β* combined with its receptors colocalized with glutamate receptor NMDA type subunit 1 (GluN1) receptors on the dorsal horn neurons increasing phosphorylation of GluN1 receptors [119]. All these molecular changes could be abolished by the depletion of the 5-HT in RVM; thus, the 5-HT_3_ receptor (neuron)-fractalkine (neuron)-IL18 (microglia)-IL1*β* (astrocytes)-GluN1 receptor (neuron) signaling pathway in dorsal horn might be the downstream mechanism of the descending pain facilitatory system [119].

Therefore, 5-HT_3_ receptors are important contributors to the production and maintenance of persistent pain via the central mechanisms in the descending pain facilitatory system. To date, randomized controlled studies have found that 5-HT_3_ receptor antagonist ramosetron can improve stool consistency, relieve abdominal pain/discomfort, and promote health-related quality of life in IBS patients [120–122], possibly based on a peripheral mechanism that blockade of 5-HT_3_ receptors in the gastrointestinal system ameliorates colonic transit rates [123]. Repeated tender point injections of 5-HT_3_ antagonist granisetron alleviated chronic myofascial pain in TMD patients [124]. 5-HT_3_ receptor antagonists dolasetron and tropisetron have been assessed for efficacy in FMS patients [125, 126]. Our recent studies also provided evidence for 5-HT_3_ receptors involved in somatic pain, an obvious symptom of FMS [127]. Specifically, we established an FMS model with three-day forced swim stress in female rats and investigated the role of the spinal 5-HT_3_ receptors in somatic pain induced by stress [127]. We found that the expression of 5-HT_3_ receptors in L4-5 spinal dorsal horn upregulated with the occurrence of pain and blocking 5-HT_3_ receptors by intrathecal administration of Y25130 reversed this process, providing a possible mechanism of 5-HT_3_ receptor antagonists applied in chronic pain without local tissue pathology [127]. As 5-HT_3_ receptor antagonists are rapidly absorbed and easily penetrate the blood-brain barrier [128], these drugs may control pain in IBS, TMD, and FMS through the central mechanisms combined with their peripheral functions, leading to a hypothesis that 5-HT_3_ receptor antagonists can be candidate drugs in chronic pain management of patients with CPP.

### 3.4. 5-HT_4_ Receptors

5-HT_4_ receptors are G protein-coupled receptors which are widely expressed in the spinal cord, whole brain, and gut [129, 130]. 5-HT_4_ receptor agonists (cisapride, mosapride, tegaserod, and prucalopride) have been widely used in IBS patients with constipation as a prokinetic medicine, mainly because of a local mechanism that presynaptic 5-HT_4_ receptors expressed in the myenteric plexus can increase peristaltic reflex activity by releasing more acetylcholine [131]. Recently, 5-HT_4_ receptor agonists have been identified with good efficacy in abdominal pain attenuation possibly based on a central mechanism. It has been found that tegaserod could reduce the rectal sensitivity to distension significantly in IBS patients [132] and the responses to nociceptive stimulation of intestine in animal models of visceral pain [133]. The analgesic effects of 5HT_4_ receptor agonists may relate to their action at a supraspinal level [20, 133]. Specifically, intravenous administration of 5-HT_4_ receptor agonists produced dose-dependent suppression of the visceromotor response (an objective measure of visceral sensation) and the activity of caudal ventrolateral medullary (CVLM) neurons, which could be blocked by intracerebroventricular pretreatment with 5-HT_4_ receptor antagonist [20], indicating the preferential involvement of supraspinal 5-HT_4_ receptors. Microinjection of tegaserod into the RVM, but not the spinal cord, attenuated the visceromotor response and reduced the spontaneous firing of lumbosacral neurons. These actions could be blocked by an intra-RVM injection of an opioid receptor antagonist or 5-HT_4_ receptor antagonist or intrathecal injection of an *α*2-adrenergic receptor antagonist, indicating that 5-HT_4_ receptors play an analgesic role through the opioidergic system in the RVM and descending noradrenergic pathways, and are excluded from the direct mechanisms at the spinal level [133]. Another study reported that intrathecal blockade of 5-HT_4_ receptors could reverse 5-HT-induced inhibition of C fiber-evoked responses on wide dynamic range neurons [134], suggesting that the spinal 5-HT_4_ receptors act in an antinociceptive role. However, in the formalin test, the activation of 5-HT_4_ receptors in the spinal cord exerted a pronociceptive effect on secondary mechanical allodynia and hyperalgesia [135]. Therefore, the facilitatory or inhibitory role of 5-HT_4_ receptors and the location in the descending pain system where they function remain to be determined. Despite the clinical application of 5-HT_4_ receptor agonists in IBS patients, the antinociceptive mechanism needs further studies.

### 3.5. 5-HT_5_ Receptors

The 5-HT_5_ receptor might be the least studied among 5-HT receptors, and very little has been learned about the functions of it. 5-HT_5A_ and 5-HT_5B_ receptors are two different subtypes which have been identified in rodents [136, 137], but in humans, only the 5-HT_5A_ receptor is functional. The human 5-HT_5B_ gene is interrupted by stop codons and transcribed and translated into nonfunctional protein [138]. Therefore, here, we only discuss 5-HT_5A_ receptors. 5-HT_5A_ receptors are highly expressed in superficial layers of the dorsal horn, lumbar dorsolateral nucleus, raphe nuclei, and higher brain areas, such as the cerebral cortex and hippocampus [139], relating to their function in psychiatric disorders (schizophrenia, bipolar disorder, anxiety, and depression) [140], memory and cognition [141], obesity [142], and pain processing [143]. To date, no 5-HT_5A_ receptor ligands have been applied to clinical practice though some selective agonists (valerenic, 5-carboxamidotryptamine) and antagonists (SB-699551) have been synthesized.

In recent years, a few studies have identified the antinociceptive role of the activation of 5-HT_5A_ receptors at the spinal level. Inspired by Doly et al.'s hypothesis that 5-HT_5A_ receptors play an analgesic role according to their distribution in the superficial layers of the spinal dorsal horn [139], Munoz-Islas and colleagues investigated their function in the formalin, capsaicin, and acetic acid writhing tests. They concluded that the antinociceptive effect induced by intrathecal injection of 5-HT or 5-carboxamidotryptamine is attributed to the activation of spinal 5-HT_5A_ receptors in both the spinal cord and DRG [143]. It has been shown that 5-HT_5A_ receptors play an analgesic role in neuropathic pain [79] and inflammatory pain [144] and contribute to antinociceptive effects of cannabinoid receptors and morphine [58]. Thus, 5-HT_5A_ receptor agonists might be a potential drug in pain relief, but further studies should be performed to examine the inhibitory function and molecular mechanisms of 5-HT_5A_ receptors in supraspinal level of descending pain modulation system.

### 3.6. 5-HT_6_ Receptors

After the 5-HT_6_ receptor was initially discovered as a novel 5-HT-sensitive receptor and its cDNA sequence identified in 1993 [145], few studies have been performed investigating its role in pain modulation. 5-HT_6_ receptors are located in the spinal cord, on GABAergic neurons of the striatum, olfactory tubercle and nucleus accumbens, and glutamatergic neurons of the hippocampus and cortex [146, 147]. Oral administration of 5-HT_6_ receptor antagonists had an antinociceptive effect in neuropathic pain either on their own or cooperating with gabapentinoids [148, 149]. Spinal injection of 5-HT_6_ receptor agonists could facilitate pain behaviors in the formalin test [150], while intrathecal treatment with 5-HT_6_ receptor antagonists decreased tactile allodynia in neuropathic pain [146]. At the supraspinal level, the activation of 5-HT_6_ receptors seems to inhibit pain, since in the DRN 5-HT_6_ receptors participated in the postictal antinociception elicited by tonic-clonic seizures [151]. However, none of study focuses on cellular or molecular mechanisms, thus why 5-HT_6_ receptors function differently in different parts of the nervous system needs further investigations.

### 3.7. 5-HT_7_ Receptors

The 5-HT_7_ receptor family is the most recently identified member of 5-HT receptors [152]. Up to date, there are three functional 5-HT_7_ subtypes in humans including 5-HT_7A_, 5-HT_7B_, and 5-HT_7D_ [153]. 5-HT_7_ receptors are expressed in the periphery and CNS of different species, including the thalamus, hypothalamus, hippocampus, striatum, cortex, and spinal dorsal horn [154]. The role of 5-HT_7_ receptors in the pain processing has been extensively studied using agonists (e.g., AS-19, LP-211, MSD-5a, and E-55888) and antagonists (e.g., SB-269970 and SB-25719). 5-HT_7_ receptors have been widely recognized to play a significant role in analgesia at the spinal level [155, 156], consistent with the immunocytochemical localization in the superficial dorsal horn and in small and medium-sized DRG cells [157]. The analgesic effect of 5-HT_7_ receptor agonists was blocked by GABA_A_ antagonists, but not GABA_B_ or opioid receptor antagonists [158], and 5-HT_7_ receptors colocalized with GABAergic cells in the dorsal horn of the spinal cord in rats with nerve injury [159], suggesting the activation of GABAergic inhibitory interneurons may account for the analgesic role of 5-HT_7_ receptors in pain processing. The blockade of 5-HT_7_ receptors reduced the antiallodynic effect of intrathecal nefopam (a nonopioid analgesic drug) or SSRIs in neuropathic pain models and abolished systemic tramadol or paracetamol-induced antinociceptive and anti-hyperalgesic effects, leading to an assumption that the spinal 5-HT_7_ receptor is a contributor in the antiallodynic efficacy of these analgesics, possibly via activating the descending serotonergic pathway and spinal 5-HT_7_ receptors [160, 161]. However, there are still some controversial results. In a neuropathic pain model, the expression of the 5-HT_7_ receptors in the spinal cord decreased and the 5-HT_7_ receptor antagonist SB-269970 evoked an analgesic effect in a dose-dependent manner in rats [162]. Although intrathecal AS-19 (agonist) reversed the increase of pain behaviors in the formalin test, neither AS-19 nor SB-269970 (antagonist) had an anti- or pronociceptive effect on carrageenan-induced mechanical allodynia [163]. These conflicting results of 5-HT_7_ receptors in the spinal cord may attribute to the use of different animal species, pain models, ligands, and detection methods.

It has been observed that 5-HT_7_ receptors play an antinociceptive role in the modulation of signals along the descending pain pathway from the supraspinal level. Spinal administration of 5-HT_7_ receptor antagonist SB-269970 blocked the antinociceptive effects of intra-RVM morphine treatment, suggesting that the descending pain inhibitory pathway from RVM act ultimately in the spinal cord in pain states through the activation of 5-HT_7_ receptors [47]. In a neuropathic pain model induced by chronic constriction injury of the sciatic nerve, the expression of 5-HT_7_ receptors in vlPAG increased, and intra-vlPAG administration of 5-HT_7_ receptor agonist AS-19 significantly and dose-dependently attenuated mechanical pain [19]. The anti-hyperalgesic effect of AS-19 could be prevented by pretreatment of SB-269970 and partially blocked by A-317491 (a P_2_X_3_ receptor antagonist), indicating that the 5-HT_7_ receptors interacting with P_2_X_3_ receptors in the vlPAG exhibit an analgesic action through the enhancement of descending pain inhibitory system [19]. Acute injection of LP-211 (a new 5-HT_7_ receptor agonist which can penetrate the blood-brain barrier) had an analgesic effect in neuropathic pain animals, which was partially mediated by an action in the ACC by targeting hyperpolarization-activated cyclic nucleotide- (HCN-) gated channels in the apical dendrites on layer 5 pyramidal cells [164]. From these studies, we can conclude that 5-HT_7_ receptors present and function along the ACC-PAG-RVM-spinal cord pathway and exert an antinociceptive effect through the descending inhibitory system; therefore, some brain-penetrant 5-HT_7_ receptor agonists can be potential candidate painkillers for pain management in the future.

The function of 5-HT_7_ receptors in CPP has been identified recently. In a morphological study, the protein level of 5-HT_7_ receptors in both the brain (hippocampus and hypothalamus) and the gut (ileum and colon) was notably higher in rats with the IBS model compared with the controls, illustrating that upregulation of 5-HT_7_ receptors in the brain and gut contributes to the pathogenesis of IBS [165]. A recent study performed on both animal models and human volunteers has confirmed the role of 5-HT_7_ receptors in IBS, showing that intestinal tissues of IBS patients and mice had higher levels of miRNA-29a but had lower levels of 5-HT_7_ receptors, and miRNA-29a knockout leads to overexpression of 5-HT_7_ receptors and attenuation in visceral hyperalgesia in IBS mice [166]. Though these two studies provide evidence of 5-HT_7_ receptors functioning in IBS both peripherally and centrally, the application of 5-HT_7_ receptor agonists in treating IBS or other CPP diseases still needs further investigations.

### 3.8. Antidepressants Affecting Serotonergic System

The commonly prescribed antidepressants affecting the serotonergic system are TCAs (e.g., desipramine, trimipramine, tianeptine, amitriptyline, doxepin, clomipramine, and imipramine), SSRIs (e.g., fluoxetine, paroxetine, citalopram, escitalopram, sertraline, and fluvoxamine), and SNRIs (e.g., venlafaxine, duloxetine, and milnacipran) [167]. The analgesic effect of these antidepressants has been long studied. The theory is that these drugs inhibit the reuptake of 5-HT and/or noradrenaline into neuronal terminals, thus leading to the accumulation of these neurotransmitters at the synaptic junction and the enhancement of pain suppression via multiple postsynaptic receptor-mediated mechanisms and the descending pain inhibitory tracts [168–170]. Among these drugs, TCAs seem to have stronger pain-relieving efficacy than SSRIs and SNRIs. For example, TCAs relieve pain in one in every 2–3 patients with neuropathic pain, while SNRIs and SSRIs are effective in one in every 4–5 and one in every 7 patients, respectively [171]. However, with lower efficacy in relieving pain, SSRIs are better tolerated and have less incidence of side effects [167]. As for CPP, these antidepressants have been examined for their efficacy in FMS, IBS, chronic headache, and low back pain.

A meta-analysis focusing on the treatment of FMS with antidepressants has shown that TCAs hold larger effect sizes for pain reduction than SSRIs [172]. The efficacy of TCAs in both pain and other symptoms (fatigue, insomnia, stiffness, and tenderness) of FMS has been well established by multiple studies, and TCAs are the first-line medication for FMS treatment [173]. Amitriptyline (10–75 mg/day) is the most frequent choice because it clinically reduces pain and sleep disturbances and its risk tolerability and safety issues at this dose are low (much lower than that in depression treatment) [174]. As for SNRIs and SSRIs, there are some positive results applied in FMS. For example, duloxetine (60-120 mg/d) had a positive effect in FMS though a greater improvement in mental symptoms rather than somatic pain may attribute to it [175]. Fluoxetine was more effective on key symptoms than placebos with good tolerability in female FMS patients at a flexible dose (45±25 mg/d) [176], and it worked better in combination with amitriptyline (20 mg fluoxetine in the morning and 25 mg amitriptyline at bedtime) [177]. However, two recent systematic reviews have shown no strong evidence supporting SNRIs and SSRIs as superior to placebo in either pain or health-related quality of life [178, 179].

Similar results for IBS in terms of the efficacy of antidepressants were reported. According to evidence-based studies, TCAs provided increased benefit for alleviating abdominal pain and improving the quality of life compared with placebo [180, 181]. Low-dose TCAs (e.g., 25 mg-75 mg/d of amitriptyline and 75 mg/d of doxepin) treatment for 1-3 months exhibited clinically significant control of IBS [182]. However, the effectiveness of SSRIs for the treatment of IBS remains controversial [180, 181, 183]. For example, six weeks of treatment with citalopram (20 mg/d for 3 weeks and 40 mg/d for the following 3 weeks) significantly suppressed IBS symptoms independent of effects on psychiatric disorders and colonic sensorimotor function in a Belgian study [184], but eight-week citalopram treatment (20 mg/d for 4 weeks and then 40 mg/d for 4 weeks) did not show superiority compared to placebo in treating nondepressed IBS patients in another study in the US [185]. As for SNRIs, several open-label pilot studies suggested that short-term duloxetine (dose ranges from 20 to 120 mg/d) is beneficial for IBS comorbid with/without depression or anxiety [186–188].

In chronic headache, a meta-analysis has illustrated that TCAs are modestly effective in relieving chronic tension-type headache [189]. Amitriptyline (25-75 mg/d) decreased headache frequency intensity and increased health-related quality of life in chronic tension headache [190], and it has been recommended as the first choice for the prophylactic treatment of this disease by the European Federation of Neurological Societies [191]. However, SSRIs and SNRIs are not supported by the evidence for the prevention of either chronic tension headache or migraine [192, 193]. As for low back pain, duloxetine (60 mg/d for about 3 months) lowered pain intensity modestly [194] but neither TCAs nor SSRIs are more effective than placebo based on previous studies [195].

We can summarize from the studies above that there is inconsistency between the efficacy of different antidepressants and different CPP conditions. In most cases, TCAs showed better results and are better choices for CPP though the side effects and adverse events always contribute to the participants' withdrawal from trials [167]. Without strong evidence in previous clinical practice, SNRIs and SSRIs should only be encouraged to apply in patients with CPP who have failed to have response to conventional therapies of TCAs or comorbid with psychiatric disorders. Still, further clinical trials with a good methodology and larger sample sizes are needed to lower the risk of bias for antidepressants.

## 4. Conclusion

Central sensitization induced by the imbalance of descending pain facilitation and inhibition systems might be a rational explanation of pain processing and comorbidities in CPP based on both experimental and clinical evidence. Treating pain as a disease rather than an individual symptom is a trend after deeper and further understanding of pathologies in the CNS. After reviewing all the studies above focusing on the role of serotonergic system in pain processing, especially in specific diseases of CPP, we conclude that either drugs targeting 5-HT receptors or antidepressants that enhance 5-HT signaling lead to a prospect of painkillers targeting this system in patients with CPP in future clinical practice. TCAs can be a good choice for several diseases of CPP including FMS, IBS, and chronic headache based on clinical evidence. The agonists of 5-HT_1A_, 5-HT_1B/1D_, 5-HT_4_, 5-HT_5A_, and 5-HT_7_ and the antagonists of 5-HT_3_ might be potential candidates of analgesic medications for CPP patients. In spite of the fact all the ligands of the above receptors have been well synthesized and examined in animal experiments, some even in humans, further investigations of their molecular mechanisms, role in the descending pain modulation system, side effects, administration methods, and effective dose and efficacy in CPP are necessary in order to provide reliable evidence for future clinical application.
